# Isolated Limb Perfusion With Melphalan Triggers Immune Activation in Melanoma Patients

**DOI:** 10.3389/fonc.2018.00570

**Published:** 2018-12-03

**Authors:** Junko Johansson, Roberta Kiffin, Annica Andersson, Per Lindnér, Peter L. Naredi, Roger Olofsson Bagge, Anna Martner

**Affiliations:** ^1^TIMM Laboratory, Sahlgrenska Cancer Center, Sahlgrenska Academy, University of Gothenburg, Gothenburg, Sweden; ^2^Department of Surgery, Institute of Clinical Sciences, Sahlgrenska Academy, University of Gothenburg, Gothenburg, Sweden; ^3^Department of Infectious Diseases, Institute of Biomedicine, Sahlgrenska Academy, University of Gothenburg, Gothenburg, Sweden; ^4^Transplantation Centre, Sahlgrenska University Hospital, Gothenburg, Sweden; ^5^Department of Surgery, Sahlgrenska University Hospital, Gothenburg, Sweden

**Keywords:** melanoma, isolated limb perfusion, melphalan, monocytes, cytotoxic T cells

## Abstract

Hyperthermic isolated limb perfusion with melphalan (M-ILP) is a treatment option for melanoma patients with metastases confined to the limbs. This study aimed at defining the role of cellular immunity for the clinical response to M-ILP in melanoma patients. It was observed that patients with enhanced cytotoxic CD8^+^ T cell reactivity to common antigens (HCMV/EBV/influenza virus) prior to M-ILP were more likely to achieve a complete disappearance of macroscopic tumors (complete response). Following M-ILP treatment, the proportions of CD16^+^ intermediate and non-classical monocytes in peripheral blood were significantly enhanced along with induction of HLA-DR on CD4^+^ and CD8^+^ T cells. For further studies of the mechanism behind melphalan-induced immune activation an *in vitro* model, aiming at mimicking the clinical M-ILP protocol, was established, where PBMCs were co-cultured with melanoma cells, which had been pre-exposed to melphalan under mild hyperthermia. Upon exposure to melphalan, melanoma cells showed increased expression of immune-related markers including MHC class I and Hsp70. Moreover, when the melphalan-treated melanoma cells were co-cultured with PBMCs, this triggered an increased proportion of CD33^+^CD14^+^CD16^++^ non-classical monocytes among the PBMCs. Furthermore, the melphalan-treated melanoma cells stimulated the expansion of CD8^+^ T cells in the co-cultured PBMCs. These cells produced enhanced levels of IFN-γ and granzyme B and were capable of killing melanoma cells. To further verify an immunogenic role of melphalan, mice were vaccinated with melphalan-exposed murine melanoma cells. When challenged with live melanoma cells, vaccinated mice showed reduced tumor growth and enhanced infiltration of tumor-specific T cells into tumors. We conclude that melphalan-exposed melanoma cells trigger expansion of CD16^+^ monocytes and activate cytotoxic T cells and that these events may contribute to the antitumoral efficacy of M-ILP.

## Introduction

For patients with recurrence of malignant melanoma, ~5–10% develop lymphatic dissemination referred to as in-transit metastasis (1). Singular lesions may be removed surgically but other treatments are warranted for patients with multiple lesions or when lesions rapidly reappear (2). Hyperthermic isolated limb perfusion with the alkylating agent melphalan (M-ILP) is a treatment option for in-transit melanoma metastases confined to a limb. During M-ILP, the affected limb is temporarily isolated from the systemic circulation and connected to a heart-lung machine. High doses of melphalan are perfused through the limb during 1 h under mild hyperthermia. During M-ILP, the concentrations of melphalan in the perfused tissue are at least 20 times higher than those achieved by the maximum tolerated dose after systemic administration (3, 4). M-ILP induces a complete response (CR), defined as a total regression of melanoma lesions in 30–80% of the patients (5, 6).

The high local concentrations of melphalan achieved during M-ILP aim to induce apoptosis of melanoma cells while sparing host cells, including immune cells, in non-perfused regions. The reconstitution of normal circulation after perfusion entails a release of tumor antigens and possibly other immunostimulatory components, including danger-associated molecular patterns (DAMPs). When these components come into contact with antigen-presenting dendritic cells (DCs), a tumor-specific T cell response likely evolve that may contribute to the response. Following M-ILP there is often a gradual regression of melanoma lesions over several months, and it can take up to 6 months before a complete response can be seen. This supports the hypothesis that part of the antitumoral effect seen by M-ILP is mediated by the immune system. This is further supported by previous research showing that melanoma patients with high counts of peripheral blood CD8^+^ T cells or high expression of the T cell activation marker HLA-DR prior to M-ILP were more likely to achieve CR (7, 8). In addition, M-ILP was reported to entail a striking induction of Melan-A specific CD8^+^ T cells in blood in a significant proportion of patients (7).

The aim of this study was to further define the role of immune effector mechanisms for the antitumoral effects of M-ILP in melanoma. The results suggest that melphalan-exposed human melanoma cells trigger an activation of the immune system, as shown by expansion of CD16^+^ monocytic cells and activation of cytotoxic T cells and that these events may contribute to the clinical efficacy of M-ILP.

## Materials and methods

### Patients and sampling of PBMCs

Twenty patients with in-transit metastasis of malignant melanoma were recruited to the study and underwent M-ILP at the Sahlgrenska University Hospital in Gothenburg, Sweden. For patients characteristics, see Table S1. Details of the ILP procedure are accounted for in earlier publications (9, 10). Briefly, under general anesthesia the blood circuit of the limb was isolated by clamping and cannulation of the major artery and vein. The cannulas were connected to an oxygenated extracorporeal unit and remaining collateral vessels were compressed with an inflatable tourniquet or an Esmarch bandage. Melphalan (Alkeran®, 13 mg/l in upper limb, 10 mg/l in lower limb) was administered into the perfusion circuit during a 20 min infusion. The temperature was held at mild hyperthermia (40°C) with a total perfusion time of 60 min. One patient also received tumor necrosis factor alpha (TNF-α, Beromun®, Boehringer Ingelheim) injected as a 1 mg bolus dose 30 min before the melphalan infusion.

Samples from peripheral blood were collected immediately before M-ILP and 1 month thereafter. For six patients, only pre-operative samples were collected. Blood cells were purified from BD Vacutainer® CPT™ cell preparation tubes (BD Biosciences, #362782) according to the manufacturer's protocol and subsequently cryopreserved. Clinical responses were evaluated according to the WHO criteria 3 months after M-ILP by the responsible physician and reported as CR (Complete Response, defined as disappearance of all lesions), PR (Partial Response, decrease of more than 50% of total tumor burden), PD (Progressive Disease, an increase of more than 25% in existing lesions or the appearance of new lesions) or SD (Stable Disease, where none of the criteria for CR, PR or PD were met). For all immunological analyses performed in this study, clinical responses were defined as either CR or Non-CR (i.e., PR, PD and SD). All patients gave written consent and the study was approved by the Regional Ethical Review Board in Gothenburg, Sweden (No. 424–14).

### Cell cultures

The human melanoma cell lines A375, SK-MEL-5, and MeWo were obtained from CLS Cell Lines Service GmbH (Eppelheim, Germany). The murine melanoma B16-F1-OVA cell line was a kind gift from Kerstin Hoffmann and Andreas Thiel (BCRT–Berlin-Brandenburg Center for Regenerative Therapies, Germany). Human cell lines were authenticated using Multiplex Cell Authentication by Multiplexion (Heidelberg, Germany) as previously described (11). The SNP profiles matched known profiles. Mycoplasma testing by PCR was performed by the Bacteriology Laboratory at the Sahlgrenska University Hospital (Gothenburg, Sweden). Cells were passaged 2–23 times after thawing before they were utilized in experiments.

A375 and SK-MEL-5 cells were cultured in DMEM (Sigma-Aldrich, #D6429) supplemented with 10% heat-inactivated fetal bovine serum (Sigma-Aldrich), 100 U/ml penicillin-streptomycin (Life Technologies, #15140122), 10 μg/ml Fungin™ (InvivoGen, #ant-fn-1), 1 mM sodium pyruvate (Laboratory medicine at Sahlgrenska University Hospital) and 2 mM L-glutamine (Life Technologies, #25030123). Human MeWo melanoma cells were cultured in DMEM/Ham's F12 (Sigma-Aldrich, #51448C) with 10% heat-inactivated fetal bovine serum, 100 U/ml penicillin-streptomycin and 10 μg/ml Fungin™. B16-F1-OVA cells were cultured in IMDM (Life-Technologies, #21980065) with 10% heat-inactivated fetal bovine serum, 100 U/ml penicillin-streptomycin, 10 μg/ml Fungin™, 1 mM sodium pyruvate and 2 mM L-glutamine. All cell lines were incubated at 37°C with 5% CO_2_.

### Antigen-specific expansion of T cells

Cryopreserved PBMCs obtained from melanoma patients before and after ILP were thawed and seeded at 2 × 10^6^ cells/ml in 96-well round bottom plates, in IMDM supplemented with 10% heat-inactivated fetal bovine serum, 100 U/ml penicillin-streptomycin and 0.6 nmol/ml antigen. Antigens were pools of peptides covering complete sequences of three melanoma-associated antigens (Peptivator® NY-ESO-1, Melan-A/MART-1, gp100/Pmel17; Miltenyi Biotec, #130-095-380, #130-094-597, #130-094-449) or peptides from HCMV/EBV/influenza virus (Peptivator® CEF MHC plus I; Miltenyi Biotec, #130-098-426) as a positive control. From day four, cultures were supplemented with 1,000 U/ml IL-2 (Proleukin®, Novartis). At day 18 or 19, expanded T cells were co-cultured with new autologous PBMCs pre-pulsed with peptides (0.6 nmol/ml), at a ratio of 1:2 (T cells:PBMC), for ~5 h in presence of Brefeldin A (Golgiplug™; BD Biosciences, # 555029). All cell cultures were kept at 37°C with 5% CO_2._ Before co-culture of new PBMCs with expanded T cells, PBMCs were stained with CellTrace™ Violet (Life Technologies, # C34557) to discriminate new PBMCs from expanded T cells in the subsequent flow cytometric analysis. In order to determine an antigen-specific response (12), a stimulation index was calculated based on the percentage of IFN-γ^+^ cells among viable CD3^+^CD8^+^Celltrace™ Violet^−^ cells (i.e., the expanded T cells) in a co-culture with T cells and peptide-pulsed PBMCs divided by the corresponding value in the co-culture with expanded T cells and unstimulated PBMCs. A stimulation index of two was set as a cutoff for a valid response against the positive control; samples with a stimulation index < 2 for the control peptide were therefore excluded from all further analysis.

### *In vitro* model of hyperthermic isolated limb perfusion

A375, MeWo and SK-MEL-5 cells were exposed to melphalan hydrochloride (Alkeran®) for 1 h at 40°C, to mimic the current clinical protocol used in M-ILP, at concentrations resulting in 20–40% cell death (50 μM for A375, 200 μM for MeWo, 60 μM for SK-MEL-5). As a hyperthermic treatment control, cells were incubated at 40°C for 1 h without cytostatic drugs, while an additional control included non-exposed, non-heat treated cells. The A375 cells were also exposed to a sub-lethal concentration (0.2 μM, causing 15–30% cell death) of daunorubicin hydrochloride (Sigma-Aldrich, #30450) for 24 h. After 24 h the melanoma cells were analyzed for immune-related stress markers by flow cytometry. Alternatively, the cells were co-cultured with PBMCs as described below.

### Co-culture of melanoma cells and PBMCs

Buffy coats from anonymous healthy donors were obtained from the blood center at the Sahlgrenska University Hospital. PBMCs were purified with dextran sedimentation followed by density gradient separation with Lymphoprep™ (Alere Technologies AS, #1114547). The PBMCs were cultured together with melphalan-exposed A375 melanoma cells in 48-wells plates with flat bottoms. After 48 h, a fraction of the PBMCs was analyzed with a myeloid panel by flow cytometry while the remaining cells were transferred to new plates for further cultivation in IMDM with 10% heat-inactivated fetal bovine serum, 100 U/ml penicillin-streptomycin, 10 μg/ml Fungin™ and 2 mM L-glutamine in the presence of 500 U/ml recombinant human IL-2 (PeproTech, #200-02) for 14 days. The expanded cells were analyzed for various T cell markers and expression of granzyme B, perforin and IFN-γ.

A portion of the expanded cells was co-incubated with fresh untreated A375 cells (CD8^+^:A375 ratio of 1:1) for 4 h followed by analysis of the degranulation of CD8^+^ T cells as reflected by surface-expression of CD107a (13). The expanded PBMCs were also co-incubated with untreated A375 (CD8^+^:A375 ratio of 0.5:1) for 27 h at 37°C in IMDM with 10% heat-inactivated fetal bovine serum and 100 U/ml penicillin-streptomycin to assess the capability of the expanded T cells to kill melanoma cells. The cytotoxicity of the T cells was assessed with an XTT cell proliferation kit (Roche, #11465015001), wherein the XTT reagent was added after 22 h and left in the culture for an additional 5 h before the absorbance was detected at 492 nm, and 690 nm for the background signal, with a FLUOstar Omega (BMG Labtech) instrument. As a control for total lysis of the melanoma cells, Triton™ X-100 (Sigma-Aldrich, #X100) was used.

### Vaccine preparation

A melphalan-based cell vaccine for an *in vivo* murine vaccination model was generated by culturing B16-F1-OVA cells in 1200 μM melphalan for 1 h. After exposure the cells were washed with a buffered sodium chloride solution and incubated overnight in fresh medium. As a negative control for immunogenic cell death, a vaccine with mitomycin C-exposed B16-F1-OVA cells was also generated (14). For the mitomycin C-based vaccine, B16-F1-OVA cells were incubated for ~20 h in medium containing 80 μM mitomycin C (Sigma-Aldrich, # M4287). Following both treatments, the cells were thoroughly washed, counted, and resuspended in a buffered saline solution. Each mouse was injected with 200–500,000 cells. Both treatments resulted in ~30–50% cell death as measured by DAPI incorporation.

### Murine vaccination model

Wild-type C57BL/6 mice (Charles River Laboratories, Germany) were inoculated on the right flank with either the melphalan- or mitomycin-based cell vaccine or saline. After 6–7 days, mice were challenged with 100,000–150,000 live B16-F1-OVA-cells injected into the left flank. Tumor growth was monitored following inoculation and experiments were terminated when any mouse achieved a tumor size of 15 mm in diameter. Tumors were then excised, weighed, processed into single cell suspensions, and analyzed by flow cytometry. To determine the number of OVA-specific T cells, cells were stained with an MHC Class I murine tetramer specific for OVA (iTAg Tetramer/PE–H-2 Kb OVA; MBL International Corporation, #TB-5001-1). Mice were all female and 8 weeks old at the start of experiments. All animal experiments were approved by the Animal Ethics Research Committee in Gothenburg, Sweden (No. 86–2014 and 195–2015).

### Flow cytometry

All flow cytometry analyzes were conducted on a BD LSRFortessa™ instrument (BD Biosciences). Antibody staining for extracellular markers was performed in phosphate buffered saline (PBS) with 0.5% BSA and 0.1% EDTA, while staining for intracellular markers was performed in permeabilization buffer (eBioscience, #00-8333-56) following fixation and permeabilisation of the cells (Fixation/Permeabilization Concentrate and Diluent; eBioscience, #00-5123-43, #00-5223-56). The conjugated antibodies are listed in Table S2. Viability of the cells was measured with a LIVE/DEAD™ Fixable Yellow, or Near IR, Dead Cell Stain Kit (Life Technologies, #L34959/#L10119) or with DAPI (Life Technologies).

### Statistics

All statistical analyses were performed in GraphPad Prism 7 (GraphPad Software). Tests on data derived from experiments involving only cell lines or murine models were parametric paired *t*-tests for single comparisons and one-way ANOVA for multiple comparisons. Other data were analyzed with non-parametric log-rank test, Mann-Whitney test or Wilcoxon matched-pairs test, except for when *n* < 5 where parametric tests were utilized instead.

## Results

### M-ILP is associated with expansion of CD16^+^ monocytes in melanoma patients

**Figure 1:**
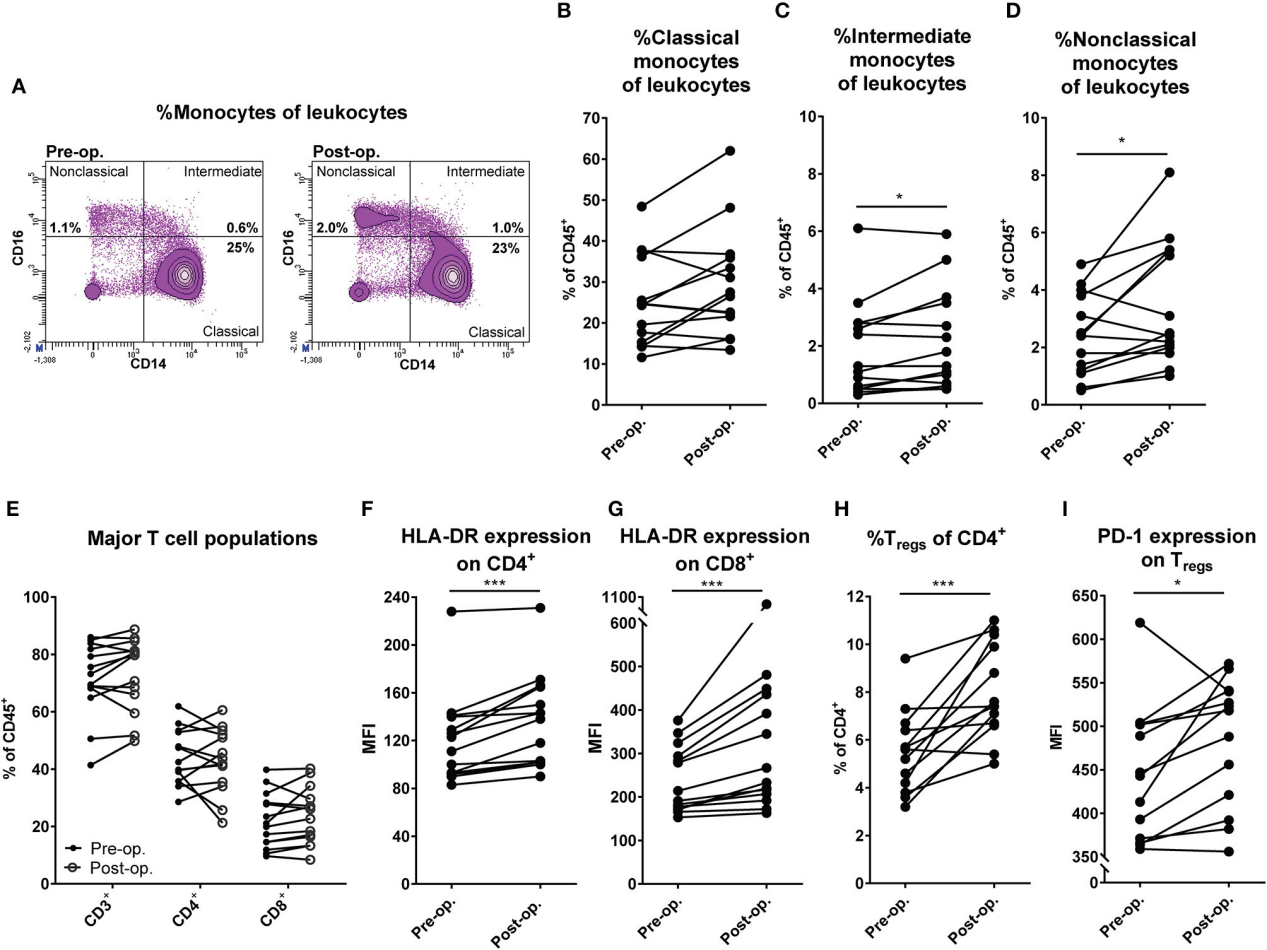
M-ILP triggers expansion of CD16^+^ monocytes and T cell activation. **(A)** Flow cytometry plots showing the gating strategy for non-classical (CD33^+^CD14^+^CD16^++^), intermediate (CD33^+^CD14^++^CD16^+^) and classical (CD33^+^CD14^++^CD16^−^) monocytes in peripheral blood before and after M-ILP in a representative patient. Percentage of **(B)** classical, **(C)** intermediate, and **(D)** non-classical monocytes among live CD45^+^ leukocytes in peripheral blood from melanoma patients before (pre-op) and 1 month after (post-op) M-ILP (*n* = 14). **(E)** Percentage of total T cells (CD3^+^), T helper cells (CD4^+^) and cytotoxic T cells (CD8^+^) among leukocytes in the peripheral blood of melanoma patients before and after M-ILP (*n* = 14). HLA-DR expression (median fluorescence intensity, MFI) on **(F)** CD4^+^ and **(G)** CD8^+^ T cells before and after M-ILP (*n* = 14). **(H)** Percentage of T_regs_ (CD3^+^CD4^+^CD25^+^Foxp3^+^) among CD4^+^ T cells and **(I)** T_reg_ expression of PD-1 before and after M-ILP (*n* = 13, Wilcoxon matched-pairs test). ^*^P ≤ 0.05, ^***^P ≤ 0.001.

Patients with in-transit melanoma metastasis confined to the limbs undergoing M-ILP treatment were recruited to the study. Peripheral blood was drawn before and 1 month after perfusion and was analyzed by flow cytometry for monocyte and DC content. There was no significant change in the fraction of classical CD14^++^CD16^−^ monocytes among leukocytes following M-ILP, while both the CD14^++^CD16^+^ intermediate and CD14^+^CD16^++^ non-classical monocyte populations increased (Figures 1A–D) (15). The frequency of CD1c^+^ DCs (CD33^+^CD14^−^HLA-DR^+^CD1c^+^), which is the most abundant DC population in blood (16), did not differ before and after M-ILP (Figures S1A,B). Seven of the 14 analyzed patients achieved CR. No significant association between CR and the induction of myeloid cell populations was observed (Figure S1C).

### T cell activation after M-ILP

To determine if M-ILP affected T cell populations, the distribution of all T cells, CD4^+^ helper T cells and CD8^+^ cytotoxic T cells in peripheral blood was analyzed before and after M-ILP for the aforementioned melanoma patients. No significant induction of these T cell populations was observed (Figure 1E). However, there was a significant increase in the level of expression of the T cell activation marker HLA-DR (17) on CD4^+^ and CD8^+^ T cells (Figures 1F,G). While all patients showed an increase in HLA-DR expression following M-ILP, the degree of induction was variable between patients. HLA-DR induction was similar in patients achieving or not achieving CR (Figure S1D). In a further analysis of T cell subpopulations it was observed that treatment with M-ILP entailed a significant expansion of regulatory T cells (T_regs_, CD3^+^CD4^+^CD25^+^Foxp3^+^) in patient blood (Figure 1H). The expanded T_regs_ showed enhanced expression of the T cell inhibitory receptor PD-1 (Figure 1I). Neither the induction of T_regs_, nor the T_reg_ PD-1 expression, prognosticated CR in this study (Figures S1E,F).

### Presence of cytotoxic T cells with specific reactivity against common antigens heralds favorable outcome of M-ILP

**Figure 2:**
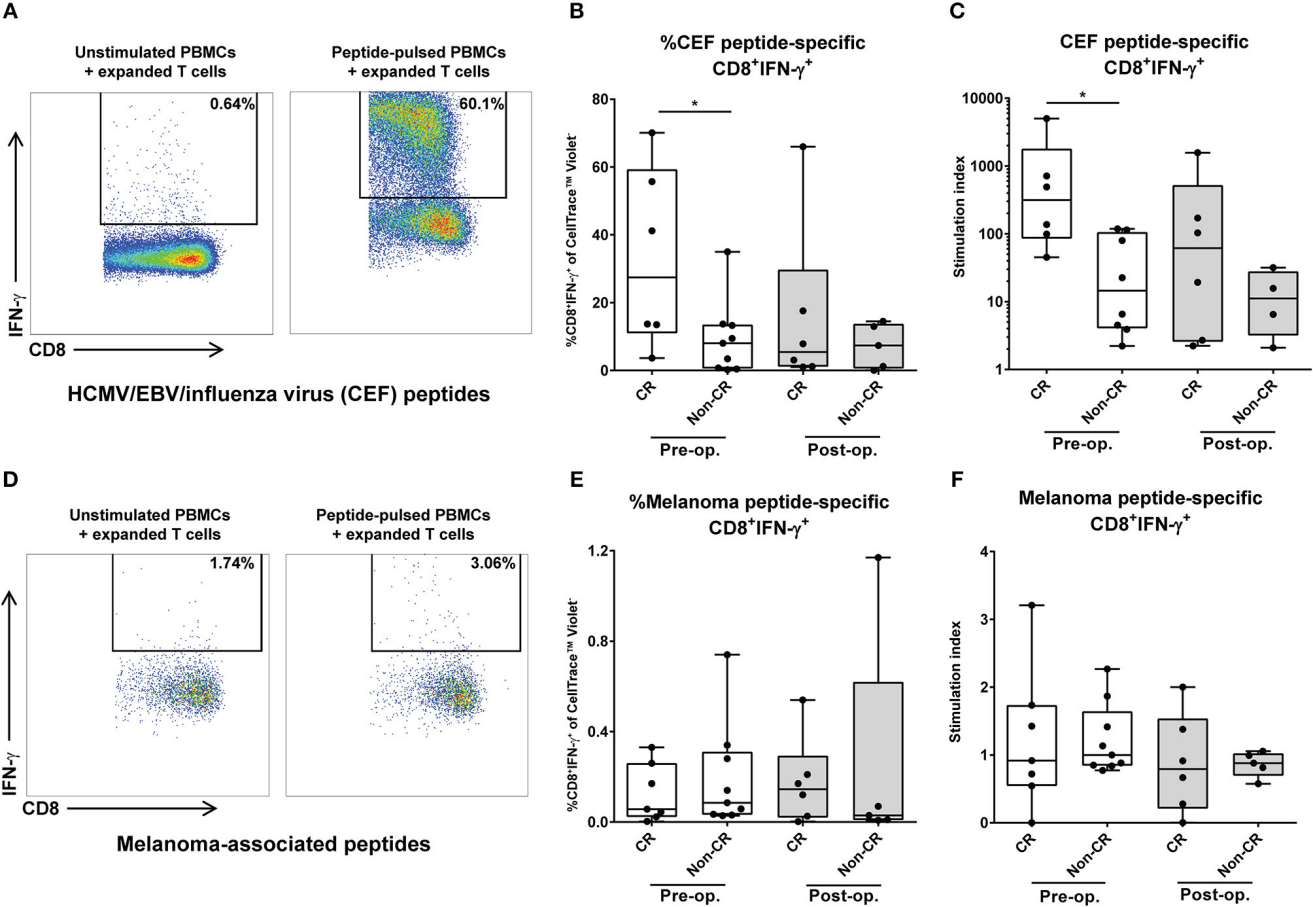
Presence of cytotoxic T cells with specific reactivity against common antigens heralds favorable outcome of M-ILP. T cells from melanoma patients undergoing M-ILP were expanded *in vitro* with **(A–C)** common antigens from HCMV/EBV/influenza virus or with **(D–F)** the melanoma-associated antigens NY-ESO-1, Melan-A/MART-1 and gp100/ in the presence of IL-2. After 18–19 days of expansion, the fraction of CD3^+^CD8^+^IFN-γ^+^ T cells responding to re-stimulation with autologous CellTrace™ Violet-stained PBMCs pulsed with the same antigens was determined. The antigen-specific stimulation index was calculated as the quotient of the percentage of viable CD3^+^CD8^+^IFN-γ^+^ cells (of CellTrace™ Violet^−^ cells) responding to peptide-pulsed PBMCs and the corresponding value in the co-culture with T cells and unstimulated PBMCs. One patient with a stimulation index < 2 for the HCMV/EBV/influenza virus peptides was excluded in all further analyses. **(A)** Flow cytometry plot showing %CD8^+^IFN-γ^+^ cells (pre-gated on viable, CellTrace™ Violet^−^CD3^+^CD8^+^ cells) from a representative patient, **(B)** %CD8^+^IFN-γ^+^ cells of the CellTrace™ Violet^−^ population, (*n* = 15 for pre-op., *n* = 11 for post-op), **(C)** antigen-specific response expressed as stimulation index, after stimulation with HCMV/EBV/influenza virus peptides (*n* = 14 for pre-op., *n* = 10 for post-op.). **(D)** Flow cytometry plot showing %CD8^+^IFN-γ^+^ cells (pre-gated on viable CellTrace™ Violet^−^CD3^+^ CD8^+^ cells), **(E)** %CD8^+^IFN-γ^+^ cells of the CellTrace™ Violet^−^ population (*n* = 16 for pre-op., *n* = 11 for post-op.), **(F)** antigen-specific response expressed as stimulation index, after stimulation with melanoma-associated peptides (*n* = 16 for pre-op., *n* = 11 for post-op.). Data are presented in box-and-whiskers plots with min. and max. Wilcoxon matched-pairs test. *P < 0.05.

To further characterize the cellular immune response in melanoma patients undergoing M-ILP, the ability of the patients' T cells to respond to specific antigens was assessed *in vitro*. PBMCs obtained before and 1 month after M-ILP were cultured with presence of peptides from common viral antigens (HCMV/EBV/influenza virus) or melanoma-associated peptides (NY-ESO-1/Melan-A/gp100), supplemented with IL-2. After 18 days of culture, the frequency of antigen-specific CD8^+^IFN-γ^+^ T cells was determined in response to re-stimulation with autologous peptide-pulsed PBMCs. At this point in time, CD3^+^ T cells constituted >95% of all cultured cells. For several of the patients, culture in the presence of common antigens (HCMV/EBV/influenza virus) triggered a robust expansion of antigen-specific cytotoxic T cells (Figures 2A–C). Patients achieving CR after M-ILP harbored a significantly higher percentage of CD8^+^IFN-γ^+^ cells (Figure 2B) and a significantly higher stimulation index (Figure 2C) in response to the common antigens prior to M-ILP, compared with patients not achieving CR. The expansion of CD8^+^ T cells to the melanoma-associated peptides (NY-ESO-1/Melan-A/gp100) was more modest (Figures 2D–F). Thus, the overall response to melanoma-associated peptides was weak in the majority of patients, with a low percentage of CD8^+^IFNγ^+^ cells (Figure 2E) and a low stimulation index (Figure 2F).

A comparison of the antigen-specific response in PBMCs sampled before M-ILP with PBMCs sampled after M-ILP did not reveal significant changes in neither the percentage CD8^+^IFN-γ^+^ cells, nor in the stimulation index over time, irrespective of antigen type (Wilcoxon matched-pairs test; Figures 2B,C,E,F).

### Melphalan-exposure induces upregulation of immune-related surface markers on melanoma cells

**Figure 3:**
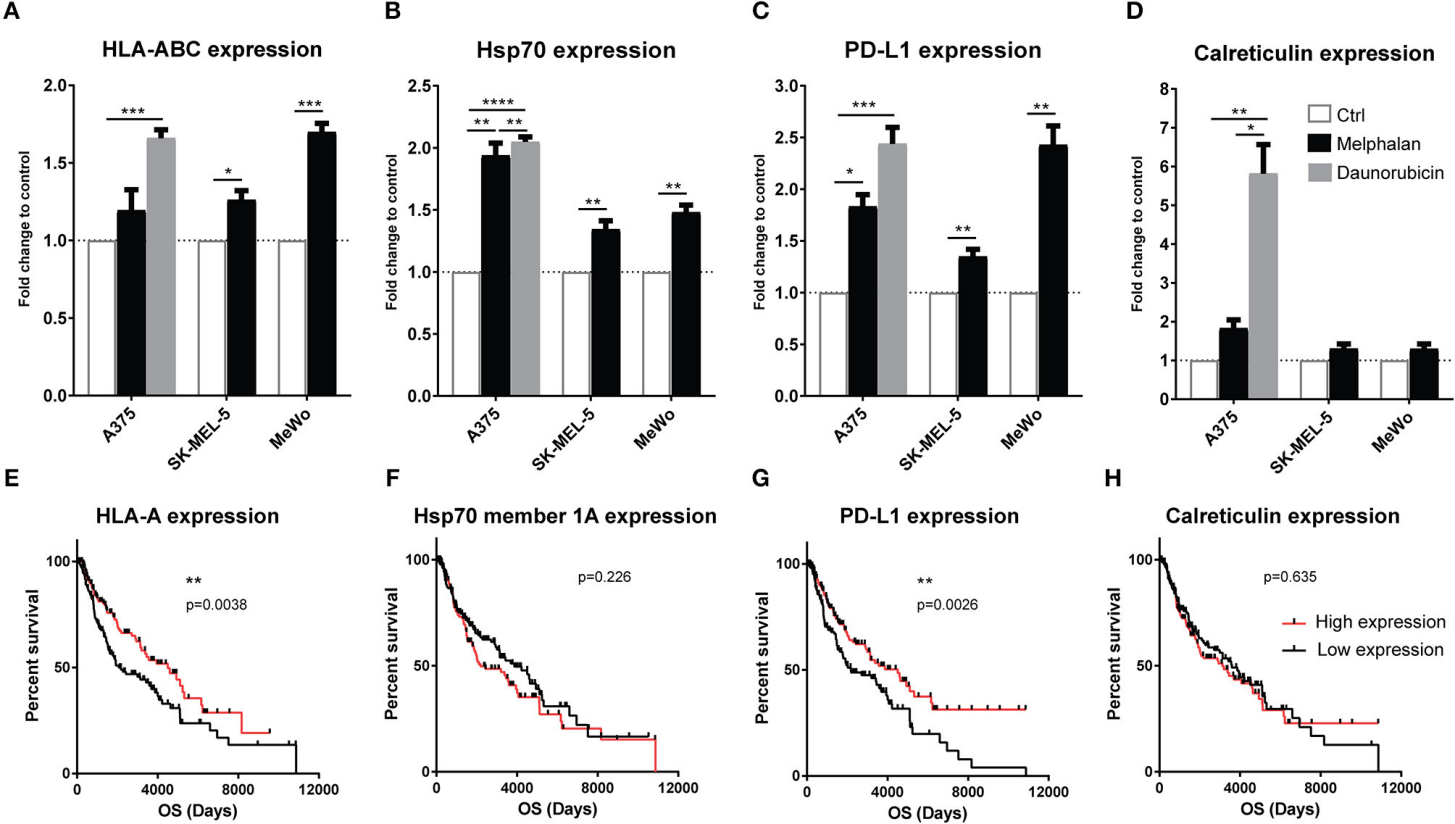
Melanoma cells exposed to melphalan upregulate immune-related surface markers. The human melanoma cell lines A375, SK-MEL-5 and MeWo were exposed to sub-lethal concentrations of melphalan for 1 h at 40°C (*n* = 6 for all cell lines). The melphalan was then washed away and the cells were cultured for an additional 24 h at 37°C. A375 cells were also exposed to a sub-lethal concentration of daunorubicin for 24 h (*n* = 5). The expression of **(A)** HLA-ABC, **(B)** Hsp70, **(C)** PD-L1, and **(D)** calreticulin was then determined by flow cytometry. The data show fold change in expression compared with non-exposed cell controls on the living populations. Paired *t*-test. Data are presented as mean values with SEM. Melanoma patients in the TCGA database were dichotomized into two groups based on above or below median gene expression of **(E)** HLA-A, **(F)** Hsp70 member 1a, **(G)** PD-L1, and **(H)** calreticulin, followed by analysis of overall survival (OS) by the log-rank test. *P ≤ 0.05, **P ≤ 0.01, ***P ≤ 0.001, ****P ≤ 0.0001.

With the aim of reproducing the clinical M-ILP procedure *in vitro*, three human melanoma cell lines (A375, SK-MEL-5, and MeWo) were exposed to melphalan for 1 h at 40°C at concentrations that triggered 20–40% cell death. Melphalan was then washed away and melanoma cells were incubated at 37°C for 24 h followed by analysis of immune-related stress markers on the living cells. In control experiments, melanoma cells were heat-treated at 40°C for 1 h in the absence of melphalan. Heat-treated melanoma cells in the absence of melphalan showed similar expression patterns as the non-exposed control cells (data not shown). The expression of MHC class I (HLA-ABC) was significantly induced on two of the cell lines by melphalan treatment, while surface-expressed Hsp70 and PD-L1 were significantly induced on all cell lines (Figures 3A–C). The expression of extracellular calreticulin, a marker of immunogenic cell death (ICD) (18, 19), was not significantly induced by melphalan in any of the cell lines (Figure 3D). Daunorubicin, which was utilized as a positive control for ICD (18), triggered robust induction of calreticulin, as well as of HLA-ABC, Hsp70 and PD-L1 in A375 melanoma cells (Figures 3A–D).

To determine the potential clinical impact of melanoma cell expression of stress-related immune markers, data from 470 melanoma patients in The Cancer Genome Atlas (TCGA, http://cancergenome.nih.gov/) were analyzed. An above-median mRNA expression of HLA-A, HLA-B, HLA-C and PD-L1 was significantly associated with overall survival (Figures 3E,G and Figures S2A–C). In contrast, the expression of calreticulin and Hsp70 genes (Hsp70 member 1a and 1b) did not significantly predict survival (Figures 3F,H and Figures S2D–E).

### Melphalan-exposed melanoma cells trigger expansion of CD16^+^ monocytes

**Figure 4:**
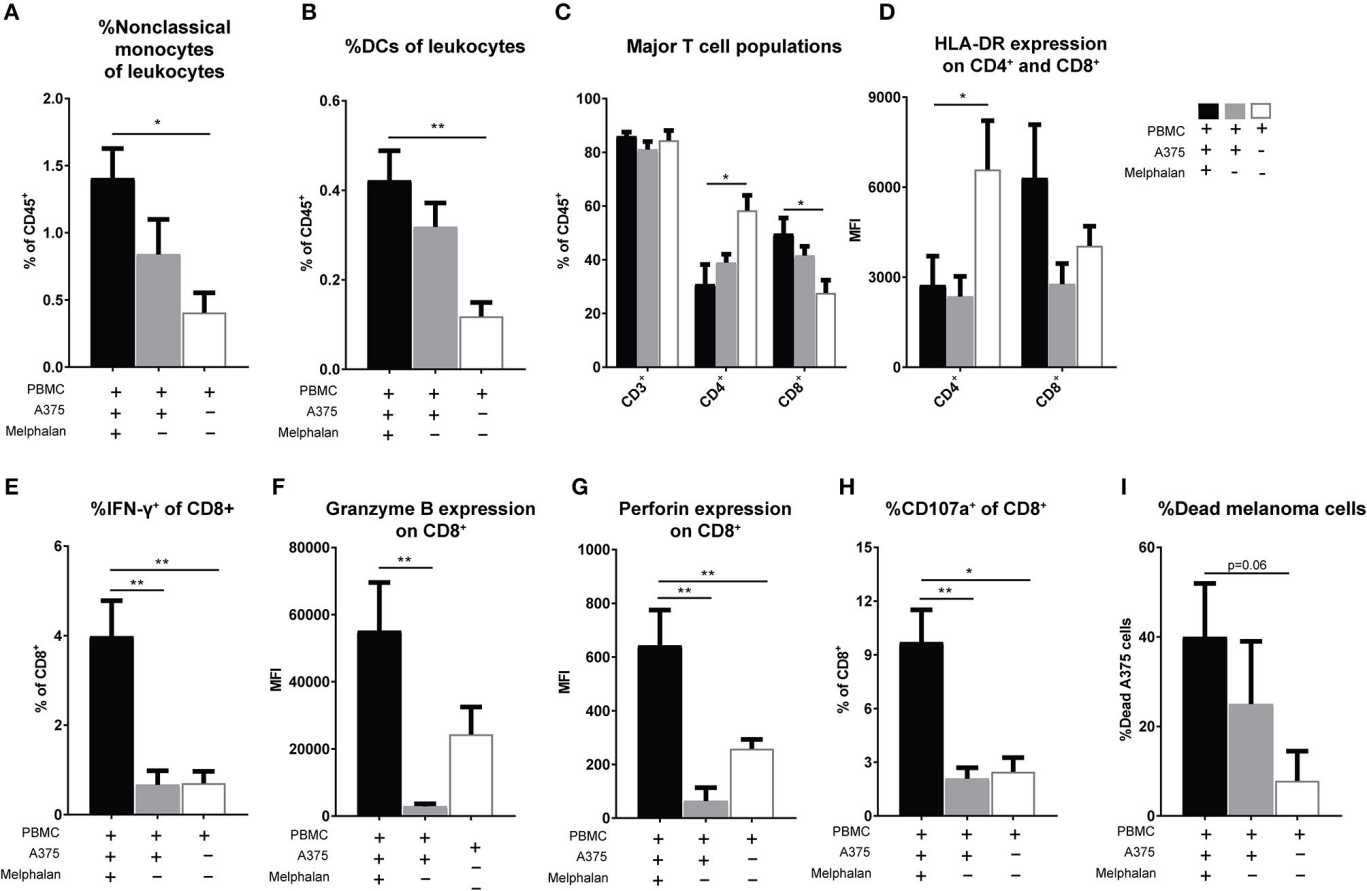
Melanoma cells exposed to melphalan induce expansion of non-classical monocytes and activation of CD8^+^ T cells. Human PBMCs were cultured alone or together with melphalan-exposed A375 cells or non-exposed A375 cells for 48 h. The percentage of **(A)** non-classical (CD33^+^CD14^+^CD16^++^) monocytes (*n* = 4 for PBMCs cultured with melanoma cells, *n* = 3 for PBMCs cultured alone, unpaired *t*-test) and **(B)** DCs (CD33^+^CD14^−^HLA-DR^+^CD1c^+^; *n* = 7 for PBMCs cultured with melanoma cells, *n* = 6 for PBMCs cultured alone, unpaired *t*-test) among live CD45^+^ leukocytes was determined by flow cytometry. After 48 h of cultivation PBMCs were further expanded for 2 weeks in the presence of IL-2. **(C)** The percentage of total T cells (CD3^+^), T helper cells (CD4^+^) and cytotoxic T cells (CD8^+^) among CD45^+^ live leukocytes were determined by flow cytometry. **(D)** The expression of HLA-DR on CD4^+^ and CD8^+^ T cells was determined (*n* = 9 for PBMCs co-cultured with melanoma cells, *n* = 8 for PBMCs cultured alone, Mann-Whitney test). The production of **(E)** IFN-γ, **(F)** granzyme B, and **(G)** perforin by CD8^+^ T cells was determined by flow cytometry (*n* = 6, Mann-Whitney test). The expanded T cells were also co-cultured with non-exposed A375 cells in a cytotoxicity assay. **(H)** The degree of degranulated CD8^+^ cells was assessed by determining the percentage of CD107a^+^ cells after 4 h of co-incubation (*n* = 5, Mann-Whitney test). **(I)** After 27 h the percentage of dead A375 cells were assessed with an XTT cell viability assay (*n* = 7, Mann-Whitney test). Data are presented as mean values with SEM. *P ≤ 0.05, **P ≤ 0.01.

To further determine effects of melphalan-exposed melanoma cells on immune cells, PBMCs were added to melphalan-exposed A375 cells 24 h after the removal of melphalan from the melanoma cells. After 2 days of co-culture, PBMCs were analyzed for monocyte and DC content by flow cytometry. As observed in patients undergoing M-ILP, the melphalan-exposed melanoma cells also triggered an increased proportion of CD33^+^CD14^+^CD16^++^ non-classical monocytes *in vitro* (Figure 4A). PBMC cultures in the presence of melanoma cells also showed an increased percentage of CD1c^+^ DCs (CD33^+^CD14^−^HLA-DR^+^CD1c^+^) among leukocytes, with a trend toward melphalan-exposed melanoma cells inducing the highest expansion of DCs (Figure 4B).

### Melphalan-exposed melanoma cells induce activation of CD8^+^ T cells

After 2 days of co-culture of melphalan-exposed melanoma cells and PBMCs, the PBMCs were transferred to a new plate and were expanded for 2 weeks in the presence of IL-2. There was a significant increase in the frequency of CD8^+^ T cells, with a corresponding decrease of CD4^+^ T cells, among the PBMCs that had been co-cultured with melphalan-exposed melanoma cells (Figure 4C). In this setting, co-culture of PBMCs with melanoma cells appeared to decrease the expression of HLA-DR on CD4^+^ T cells. However, CD8^+^ T cells from co-cultures of melphalan-treated melanoma cells tended to express higher levels of HLA-DR compared with CD8^+^ T cells from co-cultures with untreated melanoma cells (Figure 4D). Furthermore, recovered CD8^+^ T cells from the melphalan-exposed melanoma co-culture produced significantly higher levels of IFN-γ (Figure 4E) and showed elevated intracellular levels of granzyme B and perforin (Figures 4F,G). The difference was pronounced when comparing T cells from the melphalan-exposed melanoma co-culture and T cells from the non-exposed melanoma co-culture.

To determine the cytotoxic function of the expanded CD8^+^ T cells, the cells were added to untreated A375 melanoma cells in a cytotoxicity assay. After 4 h of co-incubation, CD8^+^ T cells previously exposed to melphalan-treated melanoma cells showed a significantly higher degree of degranulation, as reflected by higher CD107a expression, toward melanoma target cells (Figure 4H). Furthermore, after 27 h of co-incubation the CD8^+^ T cells from the melphalan-exposed melanoma co-culture displayed a higher capacity for killing target melanoma cells (Figure 4I).

As a control for the heat-exposure during M-ILP, melanoma cells were cultured at 40°C for 1 h in the absence of melphalan. Heat-exposure alone of melanoma cells did not affect their ability to activate myeloid cells or T cells, compared to melanoma cells cultured at 37°C (data not shown).

### Vaccination with melphalan-exposed melanoma cells induces antitumor immunity in a murine model of melanoma

**Figure 5:**
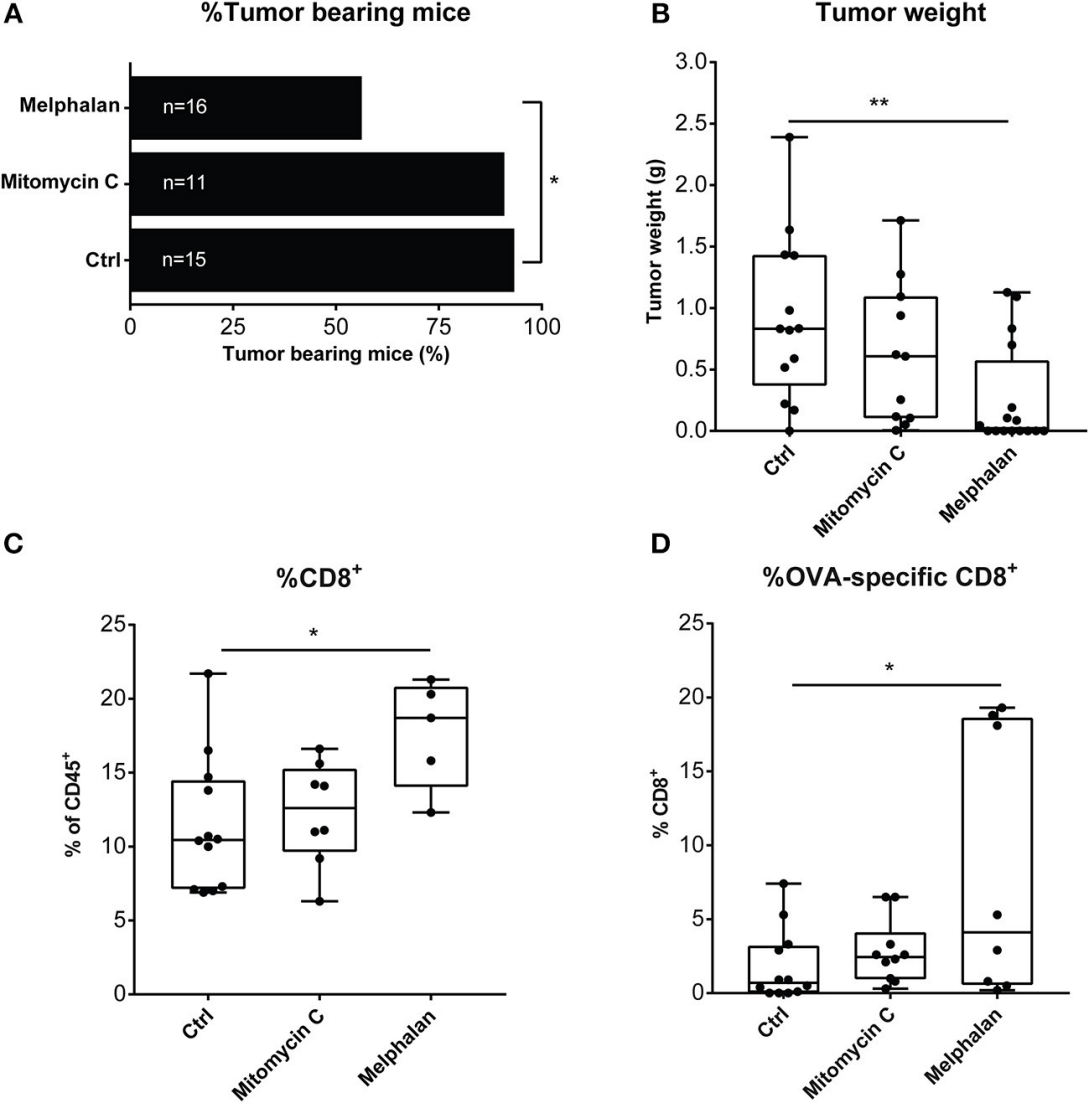
Mice treated with a melphalan-based cell vaccine are protected from tumor growth. C57BL/6 mice were vaccinated with either a mitomycin C- or melphalan-B16-F1-OVA vaccine or saline (ctrl) 6 to 7 days prior to inoculation with live B16-F1-OVA cells. **(A)** Percentage of tumor-bearing mice, **(B)** endpoint tumor weight (g), **(C)** total intratumoral CD8^+^ T cells, and **(D)** percentage of intratumoral OVA-specific T cells of CD8^+^ T cells were analyzed. Data in **(A)** are mean values presented with SD and data in, **(B–D)** are presented in box-and-whiskers plots with min. and max. One-way ANOVA. *P < 0.05, **P ≤ 0.01.

To shed further light on the potential immunogenicity of melphalan, a murine vaccination model was utilized in which C57BL/6 mice were injected with melphalan- or mitomycin C-exposed B16-F1-OVA cells in the flank to stimulate the development of protective immunity. When subsequently challenged with live melanoma cells, a significantly higher number of the mice treated with the melphalan-based vaccine failed to develop tumors compared to control mice (Figure 5A). Endpoint tumor size was also significantly reduced in the mice vaccinated with melphalan-exposed cells (Figure 5B). To determine immunological changes within the tumor that may contribute to the antitumoral response induced by the melphalan-based vaccine, the number of CD8^+^ T cells and tumor-specific CD8^+^ T cells were analyzed in the tumors. Immune priming with the melphalan-exposed cells elicited a more robust adaptive immune response compared to control and mitomycin-exposed cells as reflected by a significantly increased percentage of total intratumoral CD8^+^ T cells, and an enhanced frequency of OVA-specific CD8^+^ T cells (Figures 5C,D).

## Discussion

Hyperthermic isolated limb perfusion with melphalan is a treatment option for patients with metastatic tumors confined to a limb. The treatment is mostly used in malignant melanoma and entails CR rates of 30–80% (5, 6). After perfusion, metastases typically do not disappear until several months after treatment, thus suggesting that the cytostatic properties of melphalan are not solely responsible for the observed tumor regression, thus implying the contribution of other, yet undefined mechanisms.

For the present study, we hypothesized that T cell-mediated immunity may have contributed to the antitumor efficacy of M-ILP in patients with in-transit melanoma metastases. This assumption was based on earlier studies demonstrating that cutaneous melanoma is associated with multiple neoantigen-specific CD4^+^ and CD8^+^ T cells, presumably as the result of a high mutational burden in melanoma cells (20, 21). Local killing of metastases during M-ILP may therefore result in the release of large amounts of antigens that elicit T cell response. Additionally, the dying melanoma cells after M-ILP may express and/or release danger-associated molecular patterns (DAMPs) that potentiate immune responses as previously shown for anthracyclines, which reportedly trigger immunogenic cell death (ICD) by stimulating the DC engulfment of apoptotic bodies and by cross-presentation of antigens to cytotoxic CD8^+^ T cells (19).

In support of an immune-activating mechanism of M-ILP, we observed that human melanoma cell lines exposed to melphalan under mild hyperthermia, aiming to mimic the clinical M-ILP protocol, showed increased surface expression of the heat shock protein Hsp70 but not the “eat-me” molecule calreticulin, which accords with a previous study (22). We also observed that exposure of melanoma cells to melphalan induced expression of immune-related markers such as MHC class I (HLA-ABC), which may be relevant for the ability of CD8^+^ T cells to detect and eliminate malignant cells, and PD-L1 that serves to target PD-1 and thus may limit the T cell-mediated destruction of healthy tissue. While the implications of these findings for the clinical benefit of M-ILP remain to be determined, mining of the TCGA database revealed that high melanoma cell RNA expression of MHC class I as well as PD-L1 was associated with longer survival. No such association was seen for the ICD-related markers calreticulin and Hsp70.

Despite the absence of calreticulin induction, melphalan-exposed melanoma cells triggered protective immunity in a murine vaccination model. Hence, while injected live melanoma cells readily formed tumors in naïve mice, there were fewer measurable tumors in vaccinated mice. Tumors formed in vaccinated mice contained higher levels of CD8^+^ T cells and a higher frequency of tumor-specific CD8^+^ T cells. As a comparison to melphalan, mice were vaccinated with mitomycin C-exposed melanoma cells, prior to challenge with viable melanoma cells. While this vaccine likewise provided a degree of protective immunity, it was less efficient than the melphalan-based vaccine in preventing tumor growth and in triggering intratumoral antigen-specific T cells. These findings concur with studies demonstrating that mitomycin C is a relatively weak inducer of ICD (23).

When including PBMCs in the *in vitro* model of M-ILP, a modest but significant induction of CD16^+^ monocytes was observed. A similar induction was noted in patients undergoing M-ILP but it did not prognosticate clinical outcome. While the functional significance of the observed induction of CD16^+^ monocytes requires further investigation, the CD14^++^CD16^+^ intermediate and the CD14^+^CD16^++^non-classical monocyte populations have been shown to accumulate during infections and inflammatory conditions and to produce higher levels of cytokines such as TNF and IL-1β, compared with the classical monocytes (15). A recent study showed by single cell RNA-sequencing that while the classical and non-classical monocytes belonged to two distinct gene expression clusters (referred to as Mono1 and Mono2, respectively), the intermediate monocyte population was highly heterogeneous with cells scattered into the Mono1 and Mono2 clusters as well as into two smaller unique clusters, denoted Mono3 and Mono4 (24). The monocyte markers used in our study did, however, not allow for sub-characterization of the intermediate monocyte population. Of note, the murine counterpart of non-classical monocytes is assumed to be the Ly6C^−^CX3CR1^+^ patrolling monocytes that have been ascribed a role in reducing tumor metastasis in experimental cancer models, presumably be recruiting cytotoxic immune cells (25).

In patients and in the *in vitro* M-ILP model there were also signs of T cell activation. *In vitro* exposure of melanoma cells to melphalan triggered a pronounced induction of expanded functional CD8^+^ T cells that produced high levels of granzyme B, perforin, and IFN-γ and also degranulated in the presence of melanoma target cells in a cytotoxicity assay. In M-ILP treated melanoma patients, T cell activation was evident by increased expression of the activation marker HLA-DR on CD4^+^ and CD8^+^ T cells along with induction of regulatory T cells.

In support for an immunogenic mechanism of tumor reduction in M-ILP, patients whose CD8^+^ T cells responded strongly to a cocktail of common viral antigens (HCMV/EBV/influenza virus) were significantly more likely to achieve CR. This finding extends the results of previous studies showing that patients who achieve CR harbor T cells expressing high levels of the T cell activation marker HLA-DR prior to M-ILP (7, 8). Only low levels of melanoma-specific CD8^+^ T cells were detected in the blood of patients with no apparent differences observed between CR and non-CR patients. While the lack of detection of melanoma-specific T cell immunity may be multi-factorial, we hypothesize that activated antigen-specific CD8^+^ T cells may have entered the tumor microenvironment. In support of this hypothesis, blood drawn from mice in the vaccination study 1 week after inoculation of melphalan-treated melanoma cells showed no significant alterations in T cell populations (data not shown), while the population of intratumoral melanoma-specific CD8^+^ T cells increased significantly in mice treated with the melphalan-based cell vaccine.

From the *in vitro* M-ILP model it was obvious that the expansion of CD8^+^ T cells in PBMCs that had been co-cultured with melphalan-exposed melanoma cells was significantly enhanced by the presence of IL-2. Thus, it is possible that IL-2 treatment following M-ILP would enhance the number and cytotoxic function of T cells in melanoma patients.

It has previously been shown that the efficacy of anti-CTLA-4 therapy was pronounced for melanoma patients with high blood levels of CD16^+^ monocytes (26), and the combination of isolated limb infusion and the CTLA-4 antibody ipilimumab in a phase II trial improved response rates and was associated with an increased T cell infiltration (27). Alternatively, the efficacy of M-ILP may be boosted by anti-PD-1/PD-L1 treatment based on our results showing induction of PD-L1 on melphalan-exposed melanoma cells and induction of T_regs_ expressing high levels of PD-1.

In summary, our results lend support to the notion that the antitumor efficacy of M-ILP may, in part, be related to activation of T cell-mediated immunity. We hypothesize that the addition of IL-2, anti-CTLA-4, or anti-PD-1/PD-L1 may reinforce CD8^+^ T cell function in the tumor microenvironment to further ameliorate antitumor efficacy of M-ILP.

## Ethics statement

This study was carried out in accordance with ethics 424–14, with approval by the Regional Ethical Review Board in Gothenburg, Sweden. All subjects gave written informed consent in accordance with the Declaration of Helsinki. The protocol was approved by the Regional Ethical Review Board in Gothenburg.

Animal studies was carried out in accordance with ethics 86–2014 and 195–2015, with approval by the Animal Ethics Research Committee in Gothenburg, Sweden. The protocol was approved by the Animal Ethics Research Committee in Gothenburg.

## Author contributions

JJ designed the research, performed experiments, analyzed data, and wrote the manuscript. RK and AA performed experiments and data analysis. PL, PN, and RO designed and supervised the research and provided access to clinical samples and patient outcome data. AM conceived and supervised the project, designed the research, and wrote the manuscript. All authors read and approved the final manuscript.

### Conflict of interest statement

The authors declare that the research was conducted in the absence of any commercial or financial relationships that could be construed as a potential conflict of interest.
