# Supplementary material for: Isolated Limb Perfusion With Melphalan Triggers Immune Activation in Melanoma Patients
Source: Front Oncol. 2018 Dec 3;8:570. doi: 10.3389/fonc.2018.00570 (PMC6286961; doi:10.3389/fonc.2018.00570)
Supplement: Supplementary file 1 [file Data_Sheet_1.PDF]

*Supplementary Material*

# Isolated limb perfusion with melphalan triggers immune activation in melanoma patients

**Junko Johansson[1,2], Roberta Kiffin[1,3], Annica Andersson[1,3], Per Lindnér[2,4], Peter Naredi[2,5], Roger Olofsson Bagge[2,5] and Anna Martner[1,3*]**

[1]TIMM Laboratory, Sahlgrenska Cancer Center, Sahlgrenska Academy, University of Gothenburg, Gothenburg, Sweden

[2]Department of Surgery, Institute of Clinical Sciences, Sahlgrenska Academy, University of Gothenburg, Gothenburg, Sweden

[3]Department of Infectious Diseases, Institute of Biomedicine, Sahlgrenska Academy, University of Gothenburg, Gothenburg, Sweden

[4]Transplantation Centre, Sahlgrenska University Hospital, Gothenburg, Sweden

[5]Department of Surgery, Sahlgrenska University Hospital, Gothenburg, Sweden

*** Correspondence:**
Anna Martner
anna.martner@gu.se

## Supplementary materials

| No. | Sex | Age | Perfusion no. | No. of tumors | Largest tumor (mm) | Indication | N-Status | M-Status | Perfusion vessel | Perfusion chemo | Response |
|---|---|---|---|---|---|---|---|---|---|---|---|
| 1 | Male | 70 | 1 | 12 | 45 | Melanoma in-transit | N3 | M0 | Femoral | Melphalan | PD |
| 2 | Female | 58 | 1 | 20 | 5 | Melanoma in-transit | N2c | M0 | Femoral | Melphalan | PD |
| 3 | Female | 79 | 1 | 3 | 9 | Melanoma in-transit | N2c | M0 | Brachial | Melphalan | CR |
| 4 | Female | 86 | 1 | 6 | 10 | Melanoma in-transit | N3 | M0 | Femoral | Melphalan | CR |
| 5 | Female | 78 | 1 | 6 | 20 | Melanoma in-transit | N2c | M0 | Femoral | Melphalan | CR |
| 6 | Female | 74 | 1 | 2 | 40 | Melanoma in-transit | N2c | M0 | Brachial | Melphalan | PR |
| 7 | Male | 75 | 1 | 100 | 50 | Melanoma in-transit | N2c | M0 | Femoral | Melphalan | CR |
| 8 | Male | 66 | 1 | 30 | 10 | Melanoma in-transit | N3 | M0 | Femoral | Melphalan | CR |
| 9 | Male | 64 | 1 | 7 | 6 | Melanoma in-transit | N3 | M0 | Femoral | Melphalan | SD |
| 10 | Male | 78 | 1 | 13 | 9 | Melanoma in-transit | N3 | M0 | Femoral | Melphalan | PD |
| 11 | Female | 73 | 1 | 20 | 3 | Melanoma in-transit | N2c | M0 | Femoral | Melphalan | SD |
| 12 | Female | 60 | 1 | 23 | 5 | Melanoma in-transit | N2c | M0 | Femoral | Melphalan | PR |
| 13 | Male | 88 | 1 | 1 | 8 | Melanoma in-transit | N2c | M0 | Femoral | Melphalan | CR |
| 14 | Male | 76 | 1 | 1 | 15 | Melanoma in-transit | N2c | M0 | Brachial | Melphalan | CR |
| **15* | *Male* | *40* | *1* | *2* | *10* | *Melanoma in-transit* | *N3* | *M0* | *Femoral* | *Melphalan* | *CR* |
| **16* | *Male* | *56* | *1* | *5* | *45* | *Melanoma in-transit* | *N3* | *M0* | *Femoral* | *Melphalan* | *PD* |
| **17* | *Male* | *76* | *1* | *4* | *25* | *Melanoma in-transit* | *N3* | *M0* | *Brachial* | *Melphalan* | *PD* |
| **18* | *Female* | *52* | *1* | *4* | *20* | *Melanoma in-transit* | *N3* | *M0* | *Femoral* | *Melphalan* | *PR* |
| **19* | *Male* | *71* | *1* | *6* | *15* | *Melanoma in-transit* | *N2c* | *M0* | *Femoral* | *Melphalan* | *CR* |
| **20* | *Male* | *60* | *1* | *7* | *70* | *Melanoma in-transit* | *N2c* | *M0* | *Brachial* | *Melphalan + TNF-α* | *PD* |

**Table S1**. Patient characteristics. * For these six patients, only pre-operative samples were collected. N-status = Nodal status. M-status = Metastasis status. CR = Complete response. PR = Partial response. SD = Stable disease. PD = Progressive disease.

| **Antigen** | **Fluorophore** | **Clone** | **Vendor** | **Article number** |
|---|---|---|---|---|
| Calreticulin | PE | FMC 75 | Abcam | ab83220 |
| CD107a | PE-Cy7 | H4A3 | BD Biosciences | 561348 |
| CD14 | APC-Cy7 | MφP-9 | BD Biosciences | 333951 |
| CD16 | BV605 | 3G8 | BD Biosciences | 563172 |
| CD1c | BV421 | L161 | BioLegend | 331526 |
| CD25 | BV605 | 2A3 | BD Biosciences | 562661 |
| CD3 | BV711 | UCHT1 | BD Biosciences | 563725 |
| CD3 | FITC | HIT3a | BD Biosciences | 345763 |
| CD33 | PE-Cy7 | P67.6 | BD Biosciences | 333952 |
| CD4 | APC-H7 | RPA-T4 | BD Biosciences | 560158 |
| CD45 | BV786 | HI30 | BD Biosciences | 563716 |
| CD8 | APC | RPA-T8 | BD Biosciences | 555369 |
| CD8 | PerCP-Cy5.5 | RPA-T8 | BD Biosciences | 560662 |
| CD8 alpha | FITC | KT15 | ThermoFisher Scientific | MA516759 |
| Granzyme B | BV421 | GB11 | BD Biosciences | 563389 |
| HLA-ABC | FITC | G46-2.6 | BD Biosciences | 555552 |
| HLA-DR | FITC | L243 | BD Biosciences | 347400 |
| HLA-DR | PerCP | L243 | BD Biosciences | 347402 |
| Hsp70 | FITC | REA349 | Miltenyi Biotec | 130-105-600 |
| Foxp3 | PE | 3G3 | Miltenyi Biotec | 130-093-014 |
| IFN-γ | PE-Cy7 | B27 | BD Biosciences | 557643 |
| Perforin | FITC | δG9 | BD Biosciences | 556577 |
| PD-1 | BV421 | EH12.1 | BD Biosciences | 562516 |
| PD-L1 | PE-Cy7 | MIH1 | eBioscience | 25-5983-73 |

**Table S2**. List of all conjugated antibodies for flow cytometry utilized in the study.

## Supplementary figures

### Figure S1

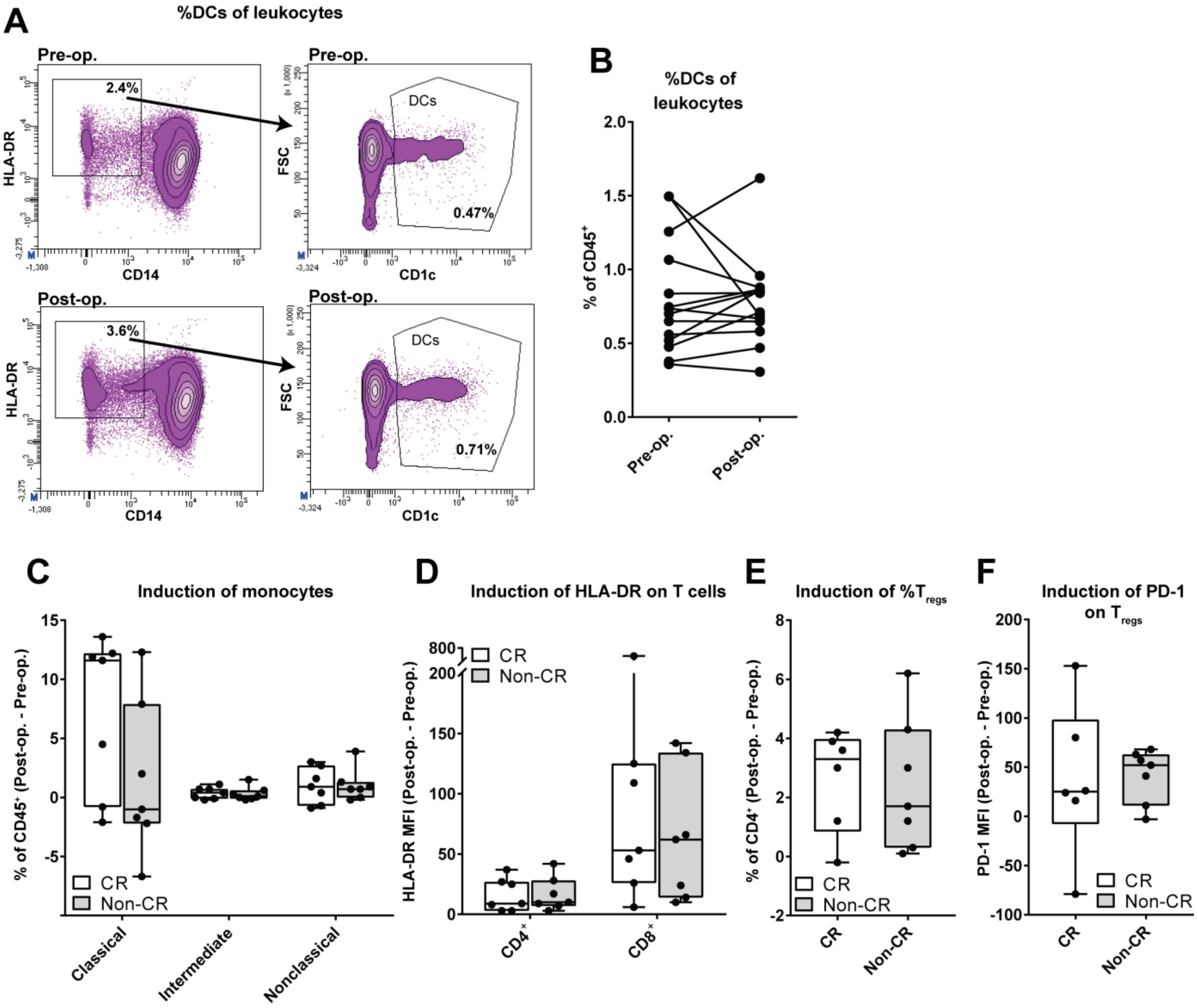


**Figure S1. Induction of myeloid cells, HLA-DR on T cells and induction of $T_{regs}$ versus clinical response after M-ILP**. **A)** Flow cytometry plots showing the gating strategy for DCs ($CD33^{+}CD14^{-}HLA\text{-}DR^{+}CD1c^{+}$) cells in peripheral blood before (pre-op) and one month after (post-op) M-ILP for a representative melanoma patient. **B)** The percentage of DCs among live $CD45^{+}$ leukocytes in peripheral blood from melanoma patients pre-op. and post-op. M-ILP (n=14, Wilcoxon matched-pairs test). Patients were stratified in two groups depending on if they achieved CR or not, and the induction (as defined by the post-op. value subtracted by the pre-op. value) of **C)** the percentage of classical ($CD33^{+}CD14^{++}CD16^{-}$), intermediate ($CD33^{+}CD14^{++}CD16^{+}$) and nonclassical ($CD33^{+}CD14^{+}CD16^{++}$) monocytes among live $CD45^{+}$ leukocytes, **D)** the expression (median fluorescence intensity, MFI) of HLA-DR on $CD4^{+}$ and $CD8^{+}$ T cells (n=14), **E)** the percentage of $T_{regs}$ ($CD3^{+}CD4^{+}CD25^{+}Foxp3^{+}$) among $CD4^{+}$ T cells and **F)** the PD-1 expression on $T_{regs}$ was determined by flow cytometry (n=13). Mann-Whitney test. Lines represent the median.

**Figure S2**

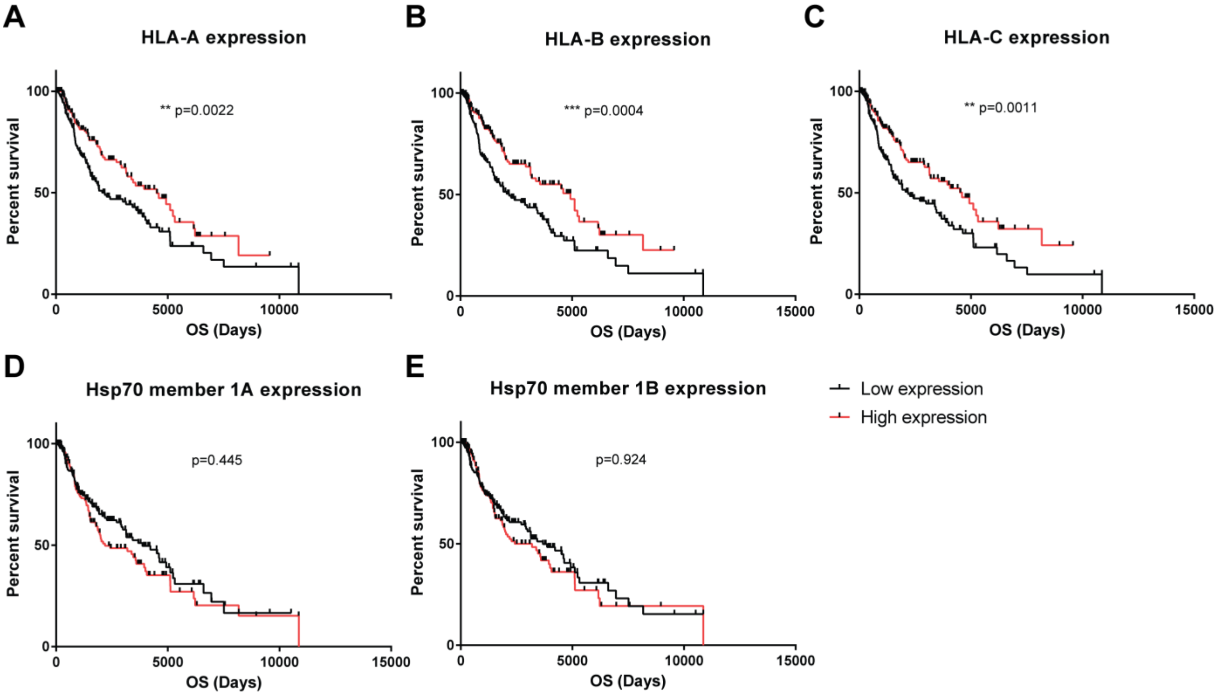


**Figure S2. Correlation between expression of immune-related genes and survival in melanoma patients.** Melanoma patients from the TCGA database were stratified into two groups based on the median gene expression levels of **A)** HLA-A, **B)** HLA-B, **C)** HLA-C, **D)** Hsp70 member 1a and **E)** Hsp70 member 1b (n=470, Log-rank test). ** P≤0.01, *** P≤0.001.
